# Relation of Local Skin Lesions to Flat Foot1Presented at the meeting of the Medical Society of the County of Erie, held June 21, 1909.

**Published:** 1909-09

**Authors:** William H. Billings

**Affiliations:** Buffalo, N. Y.


### Relation of Local Skin Lesions to Flat Foot.[1]

By WILLIAM H. BILLINGS, M. D., Buffalo, N. Y.

IN about 80 per cent. of flat foot cases, the skin covering the ankle and foot will present one or more conditions which seem to be closely related and always benefited, often cured, by correction of the deformity.

1. Presented at the meeting of the Medical Society of the County of Erie, held June 21, 1909.

Metatarsalgia or flattened transverse arch so often accompanies flat foot and is so closely related in location that it would be impossible to intelligently take up the one without the other in a subject of this kind. In the time allotted this paper it would be impossible to consider all the skin lesions associated with these deformities, so I will speak of those most commonly found and in the order of their frequency, as follows: callus, clavus or corn, excessive sweating of the feet, eczema between the toes, fissure, varicose ulcer.

Although relaxation of both arches often exists in the same foot with one or more of the above mentioned skin conditions, to prevent confusion; we will take up flat foot first, and later, the flattened transverse arch, because certain lesions seem to select locations peculiar to each deformity.

### CALLUS.

The most common skin lesion associated with flat foot is the callus. The most constant and common location is the lower internal border of the great toe. This callus may attain the size of half a hazel nut, rarely painful but has a tendency to push the great toe into hallux valgus position. This callus usually disappears with correction of the arch deformity.

The next most common site for callus formation is the dorsal surfaces of the second joints of the third, fourth and fifth toes. These are generally very painful, often present corn formation and always cured by arch correction.

### EXCESSIVE SWEATING.

Excessive sweating of the feet is a troublesome skin condition frequently met in flat foot deformity. This condition is relieved as the foot gains in strength in its corrected position.

### VARICOSE ULCER.

Varicose ulcer located posterior to and above the internal malleolus is occasionally a complication of flat foot. The correction of the deformity has hastened the healing of this to a surprising degree in four cases that have come under my observation.

### CALLUS IN FLATTENED TRANSVERSE ARCH.

In flattened transverse arch, the callus is the most constant skin manifestation. The most common location is the plantar surface of the ball of the foot and is considered of diagnostic value by some observers. The next most common site for callus formation is the external border of the foot just posterior to the fifth metatarso-phalangeal articulation.

### CORNS.

A soft corn between the fourth and fifth toes is almost always associated with transverse arch relaxation

ECZEMA BETWEEN TOES.

Eczema between the toes, although not common, is always associated with flattened transverse arch. Dr. E. Wood Ruggles, in the *Journal of Cutaneous Diseases,* published in March, 1909, contributes a long article on the relation of eczema of the toes to flattened transverse arch, and claims the flattened arch is the cause of the eczema. The location of this skin condition is usually between the third, fourth and fifth toes, and is often if not always cured by arch correction.

FISSURE.

A troublesome fissure located between the fourth and fifth toes is often associated with a relaxed transverse arch and relieved by arch correction. The skin manifestations that I have pointed out in this brief paper occur very frequently with the deformities mentioned, and the disappearance or even the improvement of them, resultant from treatment not originally intended for them, seemed an interesting subject to bring before you this evening.

1272 MAIN STREET.

